# Assessment of Knowledge, Awareness, and Practices of Human Papillomavirus Infection and Vaccination Among Medical and Dental Students: A Cross-Sectional Study

**DOI:** 10.7759/cureus.75423

**Published:** 2024-12-09

**Authors:** Sohini Saha, E Venkata Rao, Sandhya Gupta, Jyotiranjan Sahoo, Smaraki Mohanty

**Affiliations:** 1 Department of Community Medicine, Institute of Medical Sciences and Sum Hospital, Siksha 'O' Anusandhan, Bhubaneswar, IND; 2 Department of Physiology, Institute of Medical Sciences and Sum Hospital, Siksha 'O' Anusandhan, Bhubaneswar, IND

**Keywords:** awareness, cervical cancer, human papillomavirus, knowledge, practice, survey

## Abstract

Introduction

Human papillomavirus (HPV) is the most common causative agent for cervical cancer (CC) in women. Despite extensive initiatives, the acceptance and implementation of vaccinations have remained inadequate, hindering a significant impact on public health outcomes. This study aimed to provide a comprehensive assessment of the knowledge, awareness, and practices (KAP) scores of medical and dental students regarding HPV infection and vaccination.

Materials and methods

A cross-sectional online survey was conducted among 580 students, including 290 Bachelor of Dental Surgery (BDS) students and 290 Bachelor of Medicine, Bachelor of Surgery (MBBS) students. The questionnaire comprised four distinct sections: the demographic section encompassed variables including age, gender, academic year, field of study, and vaccination status. The knowledge assessment section featured eight multiple-choice inquiries, the awareness assessment incorporated eight multiple-choice items, and the practice assessment consisted of four questions using a 5-point Likert scale. Google Forms links, accompanied by a set of instructions and consent forms, were randomly dispatched via WhatsApp to the participants. Data was subjected to statistical analysis.

Results

A total of 416 responses were obtained (180 BDS and 236 MBBS), amounting to a 71.2% response rate. MBBS participants had a significantly higher mean knowledge score of 7.14 than BDS participants (6.38) (p = 0.001). Males exhibited a significantly higher mean knowledge score than females (p = 0.009). Across the study years, the highest mean knowledge score was observed among first-year students, with a significant difference among years (p = 0.001). For awareness scores, MBBS participants had a significantly higher mean score of 6.90, compared to the BDS participants of 6.36 (p = 0.001). Regarding practice, MBBS participants had a significantly higher mean score of 16.02 than BDS participants of 15.18 (p = 0.001). The study year displayed the strongest correlation with knowledge (r = 0.39), while the correlations with awareness (r = 0.13) and practice (r = 0.01) were found to be weak.

Conclusions

Low KAP scores were observed in both medical and dental students for HPV infection, CC, and vaccination.

## Introduction

Human papillomavirus (HPV) is a prevalent sexually transmitted infection with significant public health implications because of its strong association with various cancers, including cervical, oropharyngeal, anal, and penile cancers, as well as genital warts. Cervical cancer (CC), which is primarily caused by high-risk HPV strains, remains one of the leading causes of cancer-related mortality among women, particularly in low- and middle-income countries [1]. Notwithstanding the existence of highly efficacious vaccines that can avert a significant proportion of HPV-associated ailments, immunization rates continue to be lower than optimal in numerous areas [2]. The efficacy of HPV vaccination is largely contingent on the knowledge, awareness, and practices (KAP) of healthcare practitioners who play a crucial role in public awareness [3].

As future healthcare providers, medical and dental students will play a critical role in disease prevention and health promotion. Their knowledge of HPV, as well as their awareness and practices toward the HPV vaccine, directly influence their ability to educate patients, address vaccine hesitancy, and improve vaccination uptake rates. However, studies suggest that gaps in knowledge, unfavorable attitudes, and inadequate vaccination practices persist in this cohort [4]. These gaps may stem from an inadequate curricular emphasis on HPV and its vaccine or personal biases and misconceptions, which may hinder effective patient counseling in the future.

Research has consistently demonstrated that a strong recommendation from healthcare professionals is one of the most influential factors in an individual’s decision to receive an HPV vaccine [5]. Therefore, it is imperative to ensure that medical and dental students are well-informed and exhibit positive attitudes toward vaccination. This necessitates an in-depth understanding of their baseline KAP to identify areas for improvement and design-targeted educational interventions [4,5].

Given the critical role that medical and dental students play in shaping public health outcomes, evaluating their KAP toward HPV and its vaccine is essential. This study aimed to provide a comprehensive assessment of their KAP, shedding light on existing barriers to effective HPV vaccine advocacy. The findings can guide the development of educational strategies, curricular enhancements, and public health campaigns that not only equip future healthcare professionals with the requisite knowledge and skills but also foster a culture of proactive health promotion and disease prevention. By addressing these issues, this study contributes to the broader goal of reducing the global burden of HPV-related diseases by improving vaccine uptake and public awareness.

## Materials and methods

Study design and setting

This study employed a cross-sectional survey designed to assess KAP regarding HPV and its vaccine among medical and dental students. The study was conducted at the Institute of Medical Sciences and Sum Hospital, Siksha 'O' Anusandhan, Bhubaneswar, India between May 2024 and July 2024. Ethical approval was obtained from the Institutional Ethical Committee (IEC) of the Institute of Medical Sciences, SUM Hospital, Siksha 'O' Anusandhan, Bhubaneswar (IEC/IMS.SH/SOA/2024/746). Participation was voluntary, and the respondents were free to withdraw at any stage without any repercussions. Data confidentiality was maintained by anonymizing responses and storing data in password-protected systems that were accessible only to the research team. Informed consent was obtained from all participants on the first page of the online survey.

Study population

This study targeted medical and dental students enrolled in undergraduate programs. The inclusion criteria were as follows: students aged 18 years and above who were currently enrolled in medical or dental undergraduate programs, willing to participate, and provided informed consent. Students who had previously completed similar surveys and who were unwilling to provide informed consent were excluded from the study.

Sample size calculation

The sample size for this study was calculated using the Raosoft Sample Size Calculator (Raosoft Inc., Seattle, Washington, USA). Based on a 95% CI, a 3% margin of error, and an assumed population proportion of 90% awareness regarding HPV, as indicated by a reference study [6], the minimum required sample size was determined to be 377 participants. As the sample size was 377 participants, therefore, considering the response rate of 65%, the questionnaire was sent to 580 students (290 Bachelor of Dental Surgery (BDS) students and 290 Bachelor of Medicine, Bachelor of Surgery (MBBS) students).

Sampling technique

A stratified random sampling approach was employed to guarantee a proportional representation of both medical and dental students. Student demographics were categorized into strata according to their academic year and discipline of study. The participants were then randomly chosen from each stratum using a table of random numbers. Each batch consisted of 100 students.

Data collection

A systematically constructed and rigorously pre-tested questionnaire was used for data acquisition. This questionnaire was formulated based on prior empirical studies and modified to align it with a specific local context. Four experts prepared the questionnaire. Aiken's V statistic was calculated, revealing a value of 0.90, indicating a favorable level of content validity. A pilot study was conducted with 30 participants to assess the clarity and reliability of the questionnaire and determine whether the time allotted for answering it was sufficient. The study yielded a Cronbach’s alpha of 0.82 for knowledge-related questions and 0.92 for attitude and practice-related questions, indicating high internal reliability. The questionnaire comprised four distinct sections: the demographic section encompassed variables including age, gender, academic year, field of study, and vaccination status (see Appendices A, B, C, and D). The knowledge assessment section featured eight multiple-choice inquiries aimed at evaluating knowledge regarding HPV transmission, with a score of 1 given for each correct answer and 0 for each wrong answer, amounting to a maximum score of 8 and a minimum score of 0 for the category. The awareness assessment incorporated eight multiple-choice items with scoring criteria as knowledge questions. The practice assessment consisted of four questions utilizing a 5-point Likert scale, with a maximum score of 20 and a minimum score of 4. Specifically, Google Forms links, accompanied by a set of instructions and consent forms, were randomly dispatched via WhatsApp to the participants. Duplicated entries were rectified, and only fully completed responses were considered. Reminder messages were sent after one month to complete the forms. Ten minutes were given to complete the questionnaire. Google Forms does not have a direct timer; therefore, we used add-ons such as “Form Timer” to set a countdown. Once the timer ended, the form was locked for the respondent. A shorter time frame may prompt participants to focus and provide genuine, instinctive answers rather than overthinking or consulting external sources, especially for knowledge-based questions.

Statistical analysis

When participants submitted responses to a Google Form, those responses were automatically saved in a linked Google Sheet and then exported to Microsoft Excel for analysis. The analysis was done using IBM SPSS Statistics for Windows, Version 23.0 (Released 2015; IBM Corp., Armonk, NY, USA). Data distribution was assessed for normality using the Shapiro-Wilk test. Continuous variables are summarized as median, mean, and SD. Independent grouping parameters, such as gender, study group, and year of study, were compared using independent t-tests and one-way ANOVA to evaluate significant differences in the mean scores for KAP. Categorical variables, including gender and study group, are presented as frequencies and percentages. The point biserial correlation test was used for categorical data, and the Spearman correlation test was used for continuous data. The CI for the statistical tests was set at 95%.

## Results

A total of 416 (71.72%) responses were received, comprising 180 (43%) BDS and 236 (57%) MBBS students. A total of 172 (41%) patients were males and 244 (59%) were females. The study found significant variations in KAP scores across disciplines, genders, and academic years. MBBS students consistently outperformed BDS students in knowledge (7.136 vs. 6.378), awareness (6.898 vs. 6.356), and practice (16.017 vs. 15.178), all with p = 0.001. Males scored higher in knowledge than females (p = 0.009), but no gender differences were noted in awareness or practice. First-year students recorded the highest mean scores in KAP (p = 0.001). Final-year students contributed the most responses. Overall, KAP outcomes varied significantly by discipline and academic year (Table 1).

**Table 1:** Comparison of scores between grouping variables ^*^ t statistics (independent t-test) ^**^ F statistics (ANOVA test) p < 0.05: significant (S) Data presented in the form of n (%) and mean ± SD. A: awareness; BDS: Bachelor of Dental Surgery; K: knowledge; MBBS: Bachelor of Medicine, Bachelor of Surgery; P: practice

Parameter	Groups	N (%)	K score (mean ± SD)	p-value	A score (mean ± SD)	p-value	P score (mean ± SD)	p-value
Study group^*^	BDS	180 (43)	6.37 ± 1.54	0.001 (S)	6.35 ± 0.95	0.001 (S)	15.17 ± 2.16	0.001 (S)
	MBBS	236 (57)	7.13 ± 1.20		6.89 ± 1.08		16.01 ± 1.86	
Gender^*^	Male	172 (41)	7.02 ± 1.27	0.009 (S)	6.72 ± 1.02	0.355	15.69 ± 1.98	0.142
	Female	244 (59)	6.65 ± 1.48		6.62 ± 1.09		15.39 ± 2.14	
Study year^**^	First	92 (22)	7.30 ± 1.03	0.001 (S)	6.91 ± 0.88	0.001 (S)	15.82 ± 1.84	0.001 (S)
	Second	88 (21)	6.81 ± 1.44		6.90 ± 1.09		15.54 ± 1.81	
	Third	96 (23)	6.41 ± 1.41		6.16 ± 1.31		14.79 ± 2.71	
	Fourth	140 (34)	6.74 ± 1.52		6.68 ± 0.82		15.80 ± 1.77	

The study highlights gender-based disparities in KAP. Female participants outperformed males in proficient knowledge, awareness, and favorable practices. Discipline-wise, MBBS students showed higher proficiency and awareness than BDS students. Analysis by academic year revealed that knowledge and awareness peaked among fourth-year students, while the most affirmative practices were observed in first-year students. These findings suggest that gender, academic discipline, and year of study significantly influence KAP, with females and advanced MBBS students exhibiting superior outcomes (Table 2).

**Table 2:** Frequency distribution of participants for KAP based on the median score Data are presented as n (%). BDS: Bachelor of Dental Surgery; KAP: knowledge, awareness, and practices; MBBS: Bachelor of Medicine, Bachelor of Surgery

Groups	Knowledge n (%)		Awareness n (%)		Practice n (%)	
	Adequate	Inadequate	Yes	No	Good	Bad
Male	92 (22.12)	80 (19.23)	100 (24.04)	72 (17.31)	12 (2.88)	160 (38.46)
Female	152 (36.54)	92 (22.12)	144 (34.62)	100 (24.04)	172 (41.35)	72 (17.31)
BDS	96 (23.08)	84 (20.19)	152 (36.54)	28 (6.73)	132 (31.73)	48 (11.54)
MBBS	132 (31.73)	104 (25.00)	172 (41.35)	64 (15.38)	96 (23.08)	140 (33.65)
First	52 (12.50)	40 (9.62)	60 (14.42)	32 (7.69)	76 (18.27)	16 (3.85)
Second	72 (17.31)	24 (5.77)	64 (15.38)	24 (5.77)	60 (14.42)	28 (6.73)
Third	68 (16.35)	20 (4.81)	64 (15.38)	32 (7.69)	60 (14.42)	36 (8.65)
Fourth	76 (18.27)	64 (15.38)	80 (19.23)	60 (14.42)	80 (19.23)	60 (14.42)

The analysis of correlations indicated diverse associations among gender, study group, study year, and the dimensions of KAP. A moderate positive and significant correlation was identified between gender and practice (r = 0.47, p = 0.001), followed by a nonsignificant correlation between gender and knowledge (r = 0.34, p = 0.255), whereas a weak nonsignificant correlation was observed with awareness (r = 0.15, p = 0.089). The study group demonstrated a moderately significant correlation between knowledge (r = 0.27, p = 0.001) and practice (r = 0.28, p = 0.001) and a marginally weaker relationship with awareness (r = 0.25, p = 0.001). The study year displayed the strongest significant correlation with knowledge (r = 0.39, p = 0.001); correlations with awareness (r = 0.13, p = 0.009) were weak and significant, whereas correlations with practice (r = 0.01, p = 0.143) were found to be weak and nonsignificant (Table 3).

**Table 3:** Correlation analysis between independent factors and participant scores ^*^ Point biserial correlation ^**^ Spearman correlation p < 0.05: significant (S)

Parameters		Gender^*^	Study group^*^	Study year^**^
Knowledge	r	0.34	0.27	0.39
	p	0.255	0.001 (S)	0.001 (S)
Awareness	r	0.15	0.25	0.13
	p	0.089	0.001 (S)	0.009 (S)
Practice	r	0.47	0.28	0.01
	p	0.001 (S)	0.001 (S)	0.143

**Figure 1:**
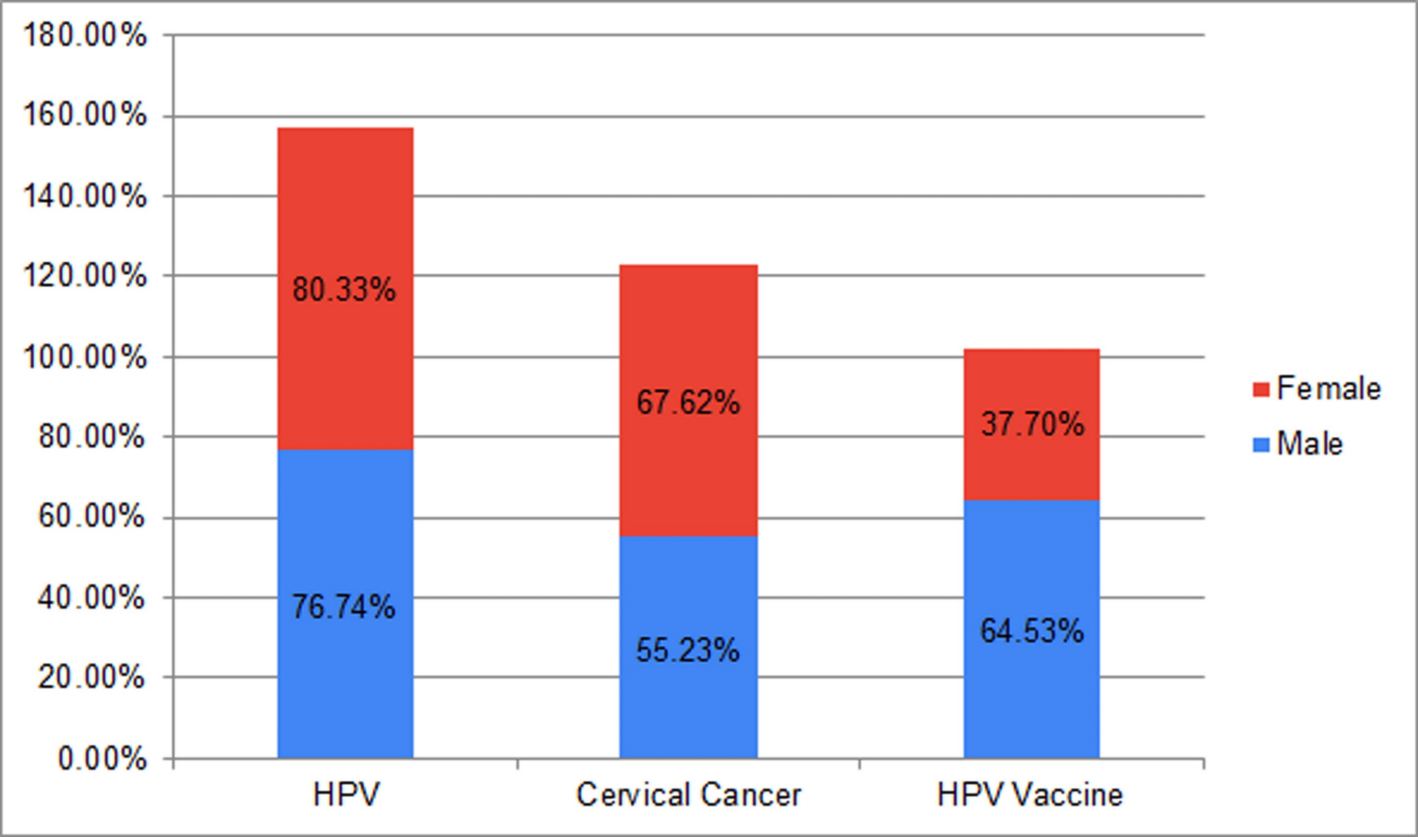
Percentage of male and female participants aware of HPV, CC, and HPV vaccine CC: cervical cancer; HPV: human papillomavirus

The level of awareness regarding HPV was notably elevated, with increased awareness in 196/244 (80.33%) females, compared to 132/172 (76.74%) males. In terms of CC awareness, 165/244 (67.62%) females exhibited marginally higher knowledge than 95/172 (55.23%) males. Awareness of the HPV vaccine reflected a comparably higher in 111/172 (64.53%) males than 92/244 (37.70%) females (Figure 1).

**Figure 2:**
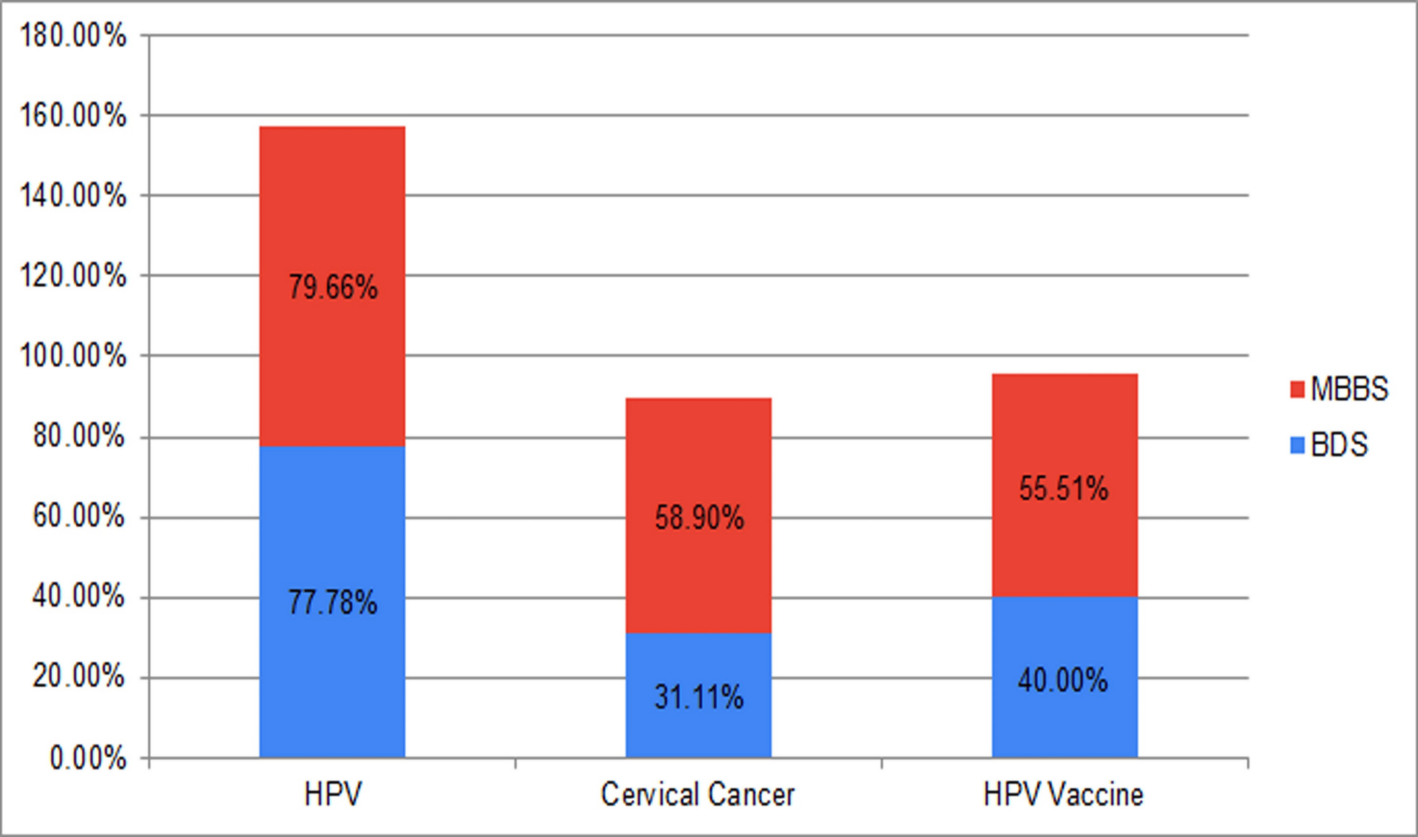
Percentage of the study groups aware of HPV, CC, and HPV vaccine CC: cervical cancer; HPV: human papillomavirus

A total of 188/236 (79.66%) MBBS students exhibited an awareness of HPV infection, for CC 139/236 (58.90%), and 131/236 (55.51%) for HPV vaccine. Conversely, BDS students demonstrated 140/180 (77.78%) awareness regarding HPV infection, for CC 56/180 (31.11%), and 72/180 (40.00%) for the HPV vaccine (Figure 2).

## Discussion

This study was conducted to provide a comprehensive evaluation of the KAP concerning HPV and HPV vaccination among students pursuing degrees in medicine and dentistry. These findings highlight the critical need for targeted interventions designed to mitigate gaps in awareness and practices among future healthcare practitioners.

Knowledge

The study revealed significant differences in knowledge scores among MBBS and BDS students, as well as across genders and academic years. MBBS students demonstrated significantly higher knowledge scores than BDS students, likely due to a more comprehensive medical curriculum. Gender differences were evident, with male students scoring higher than females, although previous research by Balaji et al. reported no such differences [7]. Notably, first-year students had the highest knowledge scores, potentially due to their recent exposure to foundational coursework. However, a decline in knowledge was observed in later academic years, underscoring the need for continuous reinforcement of HPV-related education. This aligns with the findings by Ndubuisi et al., who emphasized the importance of integrating HPV education across preclinical and clinical training [8]. To bridge knowledge gaps, curricula for both MBBS and BDS programs should include sustained HPV-related content throughout all years of study, addressing both gender and discipline disparities.

Awareness

Awareness of HPV and its vaccination was uneven across genders, disciplines, and academic years, with overall low scores reported. Female participants exhibited higher awareness of HPV and CC than males, reflecting the personal relevance of these issues. This finding is in agreement with previous studies [7,9]. However, male participants showed greater awareness of the HPV vaccine. MBBS students demonstrated slightly higher awareness scores than BDS students, emphasizing the role of medical education in fostering preventive health knowledge. In contrast, Mehta et al. [9] observed a lack of awareness regarding HPV infection among medical students. First-year students had the highest awareness scores, suggesting that early exposure to HPV topics positively influences their understanding. Previous studies, such as those by Radhika et al., also observed substantial awareness of HPV’s role in CC prevention among medical students [10]. Focused awareness campaigns targeting later academic years and both genders are crucial. Incorporating structured HPV modules into clinical training may enhance long-term retention and engagement with preventive health measures.

Practices

Despite higher knowledge and awareness, the study found generally low practice scores, with MBBS students outperforming BDS students. MBBS students exhibited better HPV-related practices than BDS students, aligning with their higher knowledge and awareness scores. This finding is in agreement with previous studies [11,12]. First-year students surprisingly recorded the highest practice scores, possibly reflecting recent vaccination campaigns or proactive attitudes among younger students. These findings contrast with studies such as Singh and Baliga, which noted better practices among clinical-year students [13]. No significant gender differences in practice scores were observed. However, the gap between knowledge/attitudes and practices highlights challenges such as limited access to vaccination services and insufficient emphasis on practical application during training [14,15]. Structural interventions such as improved access to vaccination and practical training modules are necessary to enhance HPV-related practices. Addressing barriers to implementation should be prioritized to translate knowledge and awareness into actionable healthcare practices.

Correlations

The study found moderate correlations between gender, study group, and academic year with knowledge and practices, suggesting that these factors significantly influence KAP outcomes, which is similar to previous studies [16,17]. The weak correlation between awareness and practice highlights a disconnect that needs to be addressed. Awareness campaigns alone may not suffice to improve practices, emphasizing the need for structural interventions such as accessible vaccination programs and mandatory training modules [18]. Approval of the domestically developed HPV vaccine for market entry in July facilitates the attainment of the objective of CC Elimination, which aims for a 90% vaccination rate among girls up to the age of 15 years by the year 2030 [19].

Gender disparities

The study identified notable gender disparities in HPV awareness and vaccine-related knowledge. Female participants exhibited higher awareness of HPV and CC, reflecting its greater relevance to women [20]. Conversely, male participants showed slightly higher awareness of the HPV vaccine, suggesting the need for tailored educational strategies. Similar findings were reported by Mehta et al., indicating persistent misconceptions among both genders [9].

Clinical implications

Healthcare providers with strong KAP are better equipped to educate patients about HPV, address vaccine hesitancy, and improve vaccination rates. Encouraging collaboration between medical and dental students may foster a more holistic approach to HPV-related healthcare, thus benefiting patients in diverse clinical settings. Introducing mandatory HPV-related training and reinforcing these topics in medical and dental education could ensure consistent knowledge retention and application. These findings support the need for institutional policies promoting HPV vaccination among students and faculty, serving as a model for broader community uptake [21,22]. The recommendation of introducing HPV vaccination through schools in India is a rightful and timely step in this direction [23].

Limitations

While this study provides valuable insights, several limitations must be acknowledged, such as recall bias due to self-reported data and potentially inflating KAP scores. The cross-sectional study design limits causal inferences regarding the relationship between the KAP. Longitudinal studies are needed to assess these changes over time. The findings may not be generalizable to all medical and dental students, as the study was conducted at a single institution. Multicenter studies are required for broader application. This study did not include practicing professionals, which may have limited insights into real-world practices. The differential response rate from study groups (BDS and MBBS) may have affected the KAP score. While the study identified gaps in practice, it did not extensively explore barriers such as vaccine availability, cost, or institutional support, which could further explain the observed discrepancies.

Recommendations

Based on the findings, the following recommendations are proposed: inclusion of comprehensive modules on HPV, its vaccine, and associated cancers in both medical and dental curricula, emphasizing interdisciplinary relevance. Providing hands-on training to students with practical training in patient counseling, addressing vaccine hesitancy, and administering vaccines. Establishment of on-campus vaccination clinics and subsidizing costs to encourage student uptake. Implementation of refresher courses and workshops to reinforce HPV-related knowledge and practices throughout the academic journey. Future studies should explore the barriers to HPV vaccination and assess the impact of targeted interventions on KAP outcomes.

## Conclusions

This study highlights significant disparities in KAP regarding HPV and its vaccination among medical and dental students. MBBS students demonstrated superior KAP outcomes compared to BDS students, underscoring the influence of curricular differences. Gender-based variations were evident, with males exhibiting higher knowledge but females demonstrating greater awareness of HPV and CC. First-year students recorded the highest scores in all KAP dimensions, reflecting the impact of early educational exposure. However, the low practice scores across all groups emphasize a gap between theoretical knowledge and practical application. Targeted curricular enhancements and accessible vaccination programs are essential to address these gaps and promote effective HPV prevention practices among future healthcare professionals.

## Figures and Tables

**Figure 3:**
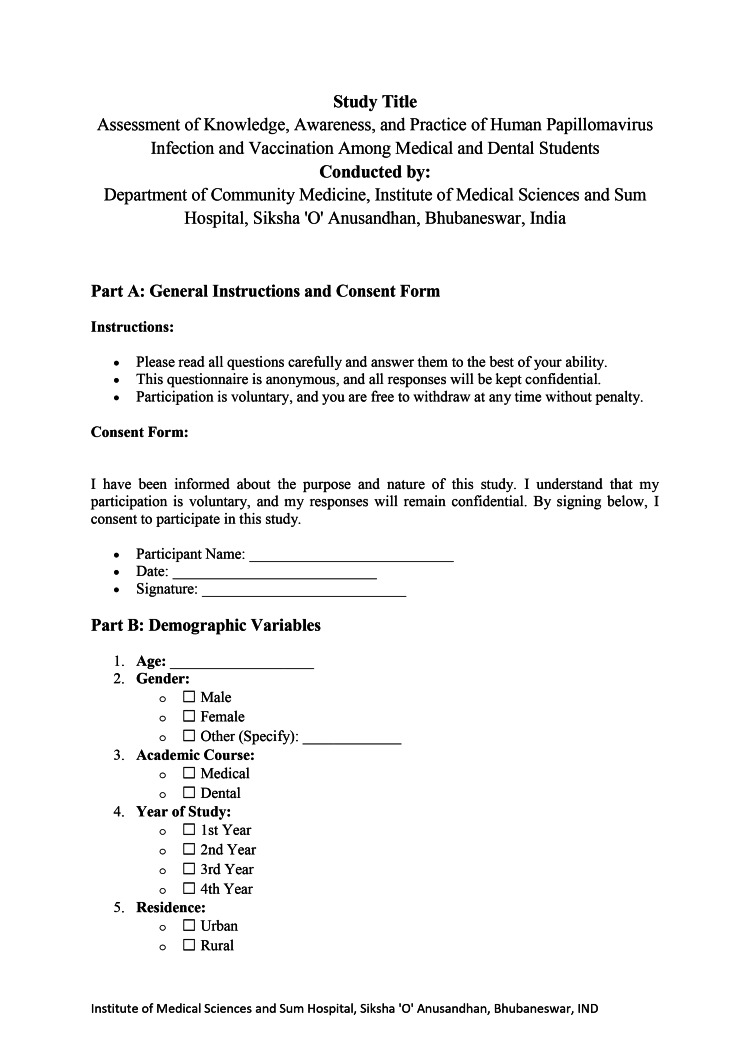
Questionnaire page 1

**Figure 4:**
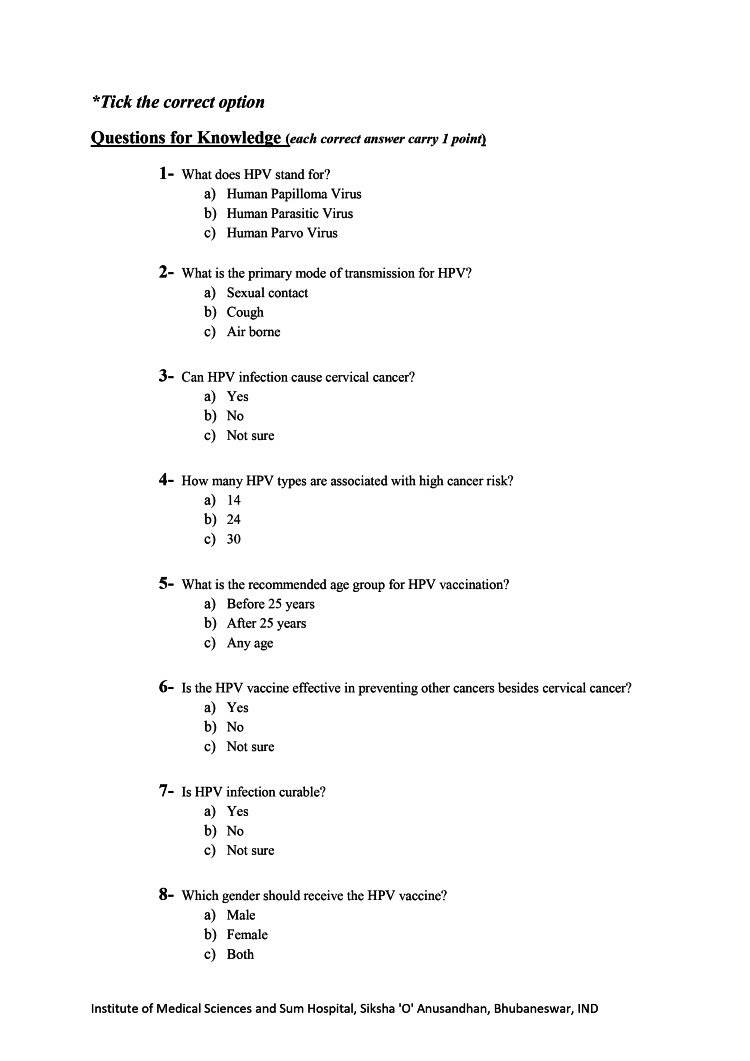
Questionnaire page 2

**Figure 5:**
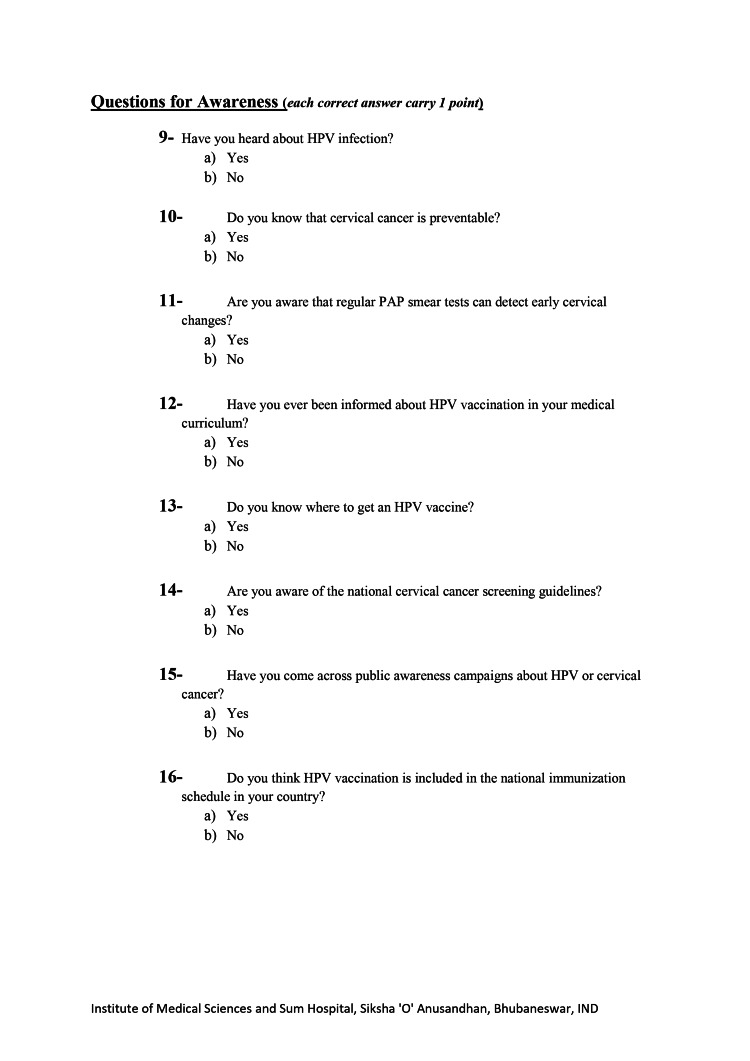
Questionnaire page 3

**Figure 6:**
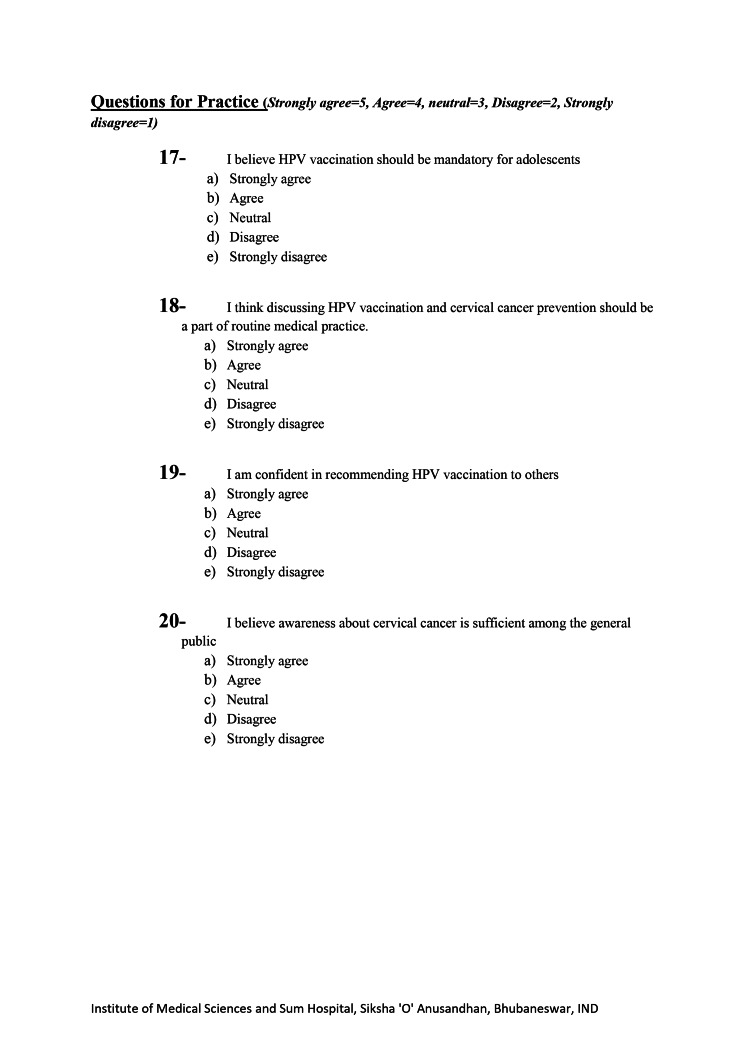
Questionnaire page 4

## Appendices

Appendix A

Appendix B

Appendix C

Appendix D
